# *Enterococcus hirae* Bacteremia Associated with Acute Pancreatitis and Septic Shock

**DOI:** 10.1155/2015/123852

**Published:** 2015-08-31

**Authors:** Peter V. Dicpinigaitis, Manuel De Aguirre, Joseph Divito

**Affiliations:** ^1^Department of Medicine, Albert Einstein College of Medicine and Montefiore Medical Center, 1825 Eastchester Road, Bronx, NY 10461, USA; ^2^Department of Radiology, Albert Einstein College of Medicine and Montefiore Medical Center, 1825 Eastchester Road, Bronx, NY 10461, USA

## Abstract

Infection with *Enterococcus hirae* has rarely been reported in humans but is not uncommon in mammals and birds. We describe a case of *Enterococcus hirae* bacteremia associated with acute pancreatitis, acute cholecystitis, and septic shock responsive to antibiotic therapy and supportive critical care management. Unique aspects of this case of *Enterococcus hirae* bacteremia are its association with acute pancreatitis and its geographical origin. To our knowledge, this is the first report of *Enterococcus hirae* bacteremia occurring in a patient in the United States. Although human infection with this organism appears to be rare, all cases reported to date describe bacteremia associated with severe and life-threatening illness. Thus, physicians need to be cognizant of the clinical significance of this heretofore little recognized pathogen.

## 1. Introduction

Infections with* Enterococcus *species including* E. faecalis* and* E. faecium* have attained great clinical significance, especially with the emergence of vancomycin-resistant strains. Human infection with* E. hirae* appears to be exceedingly rare based on published reports; however, all such cases involved bacteremia with severe illness. We herein describe a case of* E. hirae* bacteremia with unique clinical features and review the medical literature on human infection with* E. hirae*.

## 2. Case Report

An 85-year-old woman living at home was brought by ambulance to the Emergency Department (ED) with a one-day history of acute onset of nausea, vomiting, and abdominal pain. Past medical history was significant for hypertension, hyperlipidemia, and severe osteoarthritis rendering the patient wheelchair bound. She had had no recent hospitalization.

**Figure 1:**
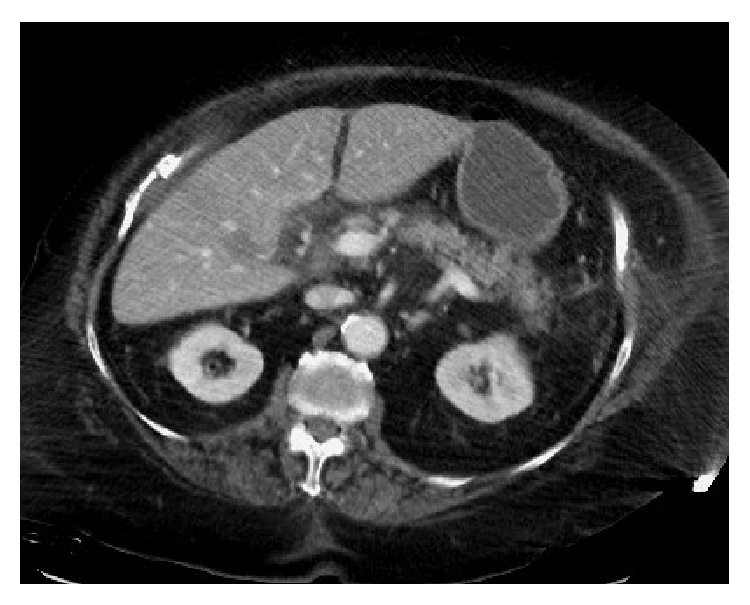
Axial CT image demonstrating inflammation of the head and body of the pancreas.

**Figure 2:**
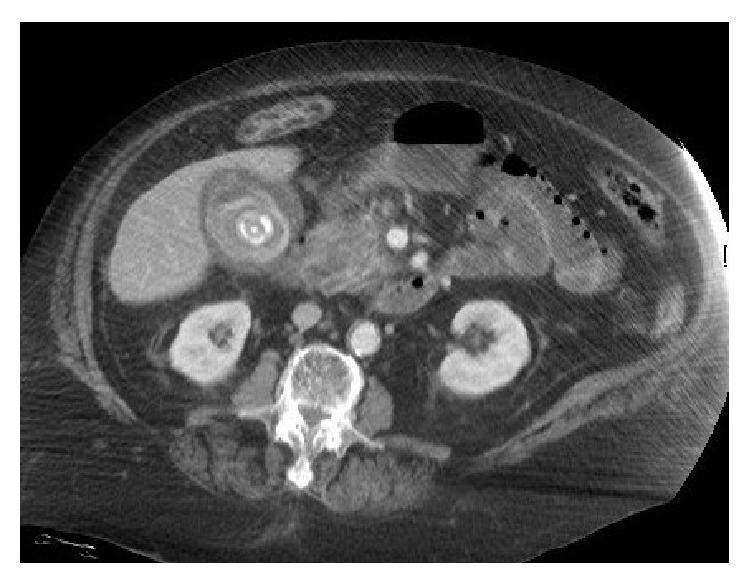
Axial CT image demonstrating gallbladder wall thickening, mucosal hyperenhancement, and a calcified gallstone.

The patient was noted to be hypotensive at her home by Emergency Medical Services and remained so on arrival to the ED (initial blood pressure 69/40 mmHg). Because of refractory hypotension despite provision of 5 liters of intravenous crystalloid, vasopressor support with norepinephrine was initiated for maintenance of adequate blood pressure. Initial laboratory evaluation demonstrated white blood cells, 4.8 × 10^9^/liter; hemoglobin, 12.3 g/dL; platelets, 301 × 10^9^/liter; blood urea nitrogen (BUN), 12 mg/dL; creatinine, 1.06 mg/dL; total bilirubin, 2.0 mg/dL; direct bilirubin, 1.9 mg/dL; alkaline phosphatase, 239 U/L; SGOT, 428 U/L; SGPT, 125 U/L. The presence of acute pancreatitis was indicated by elevated serum lipase, 2283 U/L, and amylase, 757 U/L, and was confirmed by computed tomography (CT) of the abdomen (Figure 1). In addition, the CT scan demonstrated gallbladder wall thickening, mucosal hyperenhancement, and multiple calcified gallstones, consistent with acute cholecystitis (Figure 2). Empiric antibiotic therapy was begun with intravenous vancomycin, 1 gram every 12 hours, and piperacillin/tazobactam, 4.5 mg every 8 hours. Worsening respiratory distress necessitated intubation and initiation of mechanical ventilation in the ED before transfer to the intensive care unit (ICU) for further evaluation and management.

In the ICU, echocardiogram confirmed normal left and right ventricular size and systolic function. Addition of phenylephrine and vasopressin infusions to norepinephrine was required for maintenance of adequate blood pressure in the setting of refractory, vasodilatory septic shock. Piperacillin/tazobactam was discontinued and empiric therapy with cefepime, 1 gram every 12 hours, was initiated. Abdominal ultrasound demonstrated a nondilated common bile duct. Two sets of blood cultures drawn at the time of admission (each set consisted of one BD BACTEC plus aerobic/F culture vial and one BD BACTEC plus anaerobic/F culture vial) subsequently grew* Enterococcus hirae* in one of the two sets and* Klebsiella pneumoniae* in both sets (final identification by MALDI-TOF MS).* E. hirae* was found sensitive to ampicillin, vancomycin, and ciprofloxacin.* K. pneumoniae* was sensitive to cefazolin, cefepime, gentamicin, ciprofloxacin, and trimethoprim/sulfamethoxazole and resistant to ampicillin and ampicillin/sulbactam. Repeat blood cultures drawn on the second hospital day remained negative. Based on sensitivities, antibiotic therapy was changed on hospital day 8 to ceftriaxone, 1 gram every 24 hours, and ampicillin, 1 gram every 6 hours, to complete a 14-day course of treatment. Surgical intervention was deferred because of the patient's response to antibiotics and other supportive therapies. Percutaneous cholecystostomy was considered but was precluded by the presence of multiple large gallstones. The patient was eventually weaned off vasopressor support on hospital day 7, extubated and removed from mechanical ventilation on hospital day 10, and discharged to home on hospital day 15 having completed 2 weeks of intravenous antibiotic therapy.

## 3. Discussion

Human infection with* Enterococcus hirae* appears to be exceedingly rare, though not uncommon in mammals and birds. Indeed, a National Library of Medicine (PubMed) search performed in April 2015 revealed only 8 publications describing 9 patients with* E. hirae* infection, all involving bacteremia [1–8] and associated with a variety of clinical conditions (Table 1). A retrospective study of 1887 cases of enterococcal bacteremia from one institution in Taiwan revealed 0.5% of isolates to be* E. hirae* [9]. A recent report documented the first isolation of* E. hirae* from human umbilical cord blood [10].

The case presented herein is unique in its association with acute pancreatitis, though one such report may be found in the veterinary literature [11]. Furthermore, our patient is the first to be reported from the United States; one previous North American case has been reported from Canada [3]. Septic shock in association with* E. hirae* bacteremia, as in our patient, has been previously described as a complication of spontaneous bacterial peritonitis [7] and of pyonephrosis [8]. Our case also appears to be the first to report simultaneous bacteremia with a second organism,* Klebsiella pneumoniae*. However, one previous publication describes the occurrence of* Lactococcus garvieae* bacteremia one month prior to demonstration of* E. hirae* bacteremia in a patient with native valve bacterial endocarditis [3]. In the present case, simultaneous bacteremia with* K. pneumonia* may indeed have contributed in a significant fashion to the severity of the patient's illness, as this organism has been associated with severe acute pancreatitis [12], though few such cases have been reported. Furthermore, bacteremia with* K. pneumonia* from a variety of other sources has been clearly associated with the occurrence of septic shock [13].

Human infection with* E. hirae* continues to emerge as a source of severe and life-threatening illness. All reported cases to date describe bacteremia [1–8], in association with a variety of clinical conditions including endocarditis [2, 3, 5], acute pyelonephritis [6, 8], acute cholecystitis [6], and spontaneous bacterial peritonitis [7], with some cases progressing to septic shock [7, 8]. Health care providers need to be aware of the clinical significance of this heretofore little recognized pathogen.

## Figures and Tables

**Table 1:** Published cases of human *Enterococcus hirae* bacteremia.

Age	Sex	Country	Associated condition(s)	Reference
49	M	Israel	End-stage renal disease/hemodialysis	[1]
72	M	France	Native aortic-valve endocarditis	[2]
80	M	Canada	Native aortic-valve endocarditis	[3]
55	M	Spain	Spondylodiscitis/epidural and psoas abscess	[4]
78	F	France	Prosthetic aortic-valve endocarditis (relapse)	[5]
62	F	Taiwan	Acute pyelonephritis	[6]
86	F	Taiwan	Acute cholecystitis	[6]
61	M	Korea	Cirrhosis/spontaneous bacterial peritonitis	[7]
44	M	France	Cirrhosis/pyonephrosis	[8]

## References

[1] Gilad J., Borer A., Riesenberg K., Peled N., Shnaider A., Schlaeffer F. (1998). *Enterococcus hirae* septicemia in a patient with end-stage renal disease undergoing hemodialysis. *European Journal of Clinical Microbiology & Infectious Diseases*.

[2] Poyart C., Lambert T., Morand P. (2002). Native valve endocarditis due to *Enterococcus hirae*. *Journal of Clinical Microbiology*.

[3] Vinh D. C., Nichol K. A., Rand F., Embil J. M. (2006). Native-valve bacterial endocarditis caused by *Lactococcus garvieae*. *Diagnostic Microbiology and Infectious Disease*.

[4] Canalejo E., Ballesteros R., Cabezudo J., García-Arata M. I., Moreno J. (2008). Bacteraemic spondylodiscitis caused by *Enterococcus hirae*. *European Journal of Clinical Microbiology and Infectious Diseases*.

[5] Talarmin J. P., Pineau S., Guillouzouic A. (2011). Relapse of *Enterococcus hirae* prosthetic valve endocarditis. *Journal of Clinical Microbiology*.

[6] Chan T.-S., Wu M.-S., Suk F.-M. (2012). *Enterococcus hirae*-related acute pyelonephritis and cholangitis with bacteremia: an unusual infection in humans. *Kaohsiung Journal of Medical Sciences*.

[7] Sim J. S., Kim H. S., Oh K. J. (2012). Spontaneous bacterial peritonitis with sepsis caused by *Enterococcus hirae*. *Journal of Korean Medical Science*.

[8] Brulé N., Corvec S., Villers D., Guitton C., Bretonnière C. (2013). Life-threatening bacteremia and pyonephrosis caused by *Enterococcus hirae*. *Medecine et Maladies Infectieuses*.

[9] Tan C.-K., Lai C.-C., Wang J.-Y. (2010). Bacteremia caused by non-faecalis and non-faecium enterococcus species at a medical center in Taiwan, 2000 to 2008. *Journal of Infection*.

[10] Savini V., Bonfini T., Marrollo R. (2014). *Enterococcus hirae*: a zoonotic microorganism in human umbilical cord blood. *World Journal of Microbiology and Biotechnology*.

[11] Lapointe J. M., Higgins R., Barrette N., Milette S. (2000). *Enterococcus hirae* enteropathy with ascending cholangitis and pancreatitis in a kitten. *Veterinary Pathology*.

[12] Tugal D., Lynch M., Hujer A. M., Rudin S., Perez F., Bonomo R. A. (2015). Multi-drug-resistant *Klebsiella pneumonia* pancreatitis: a new challenge in a serious surgical infection. *Surgical Infections*.

[13] Togawa A., Toh H., Onozawa K. (2015). Influence of the bacterial phenotypes on the clinical manifestations in *Klebsiella pneumoniae* bacteremia patients: a retrospective cohort study. *Journal of Infection and Chemotherapy*.

